# Effect of myocardial dysfunction in cardiac morbidity and all cause mortality in childhood cancer subjects treated with anthracycline therapy

**DOI:** 10.1186/s40959-015-0005-8

**Published:** 2015-11-26

**Authors:** Olga H. Toro-Salazar, Eileen Gillan, Joanna Ferranti, Andrea Orsey, Karen Rubin, Shailendra Upadhyay, Wojciech Mazur, Kan N. Hor

**Affiliations:** 1 Connecticut Children’s Medical Center, 282 Washington Street, Hartford, CT 06106 USA; 2 Ohio Heart and Vascular Center, The Christ Hospital, Cincinnati, OH USA; 3 Nationwide Children’s Hospital, Columbus, OH USA

**Keywords:** Anthracyclines, Cardiotoxicity, Cardiomyopathy

## Abstract

**Background:**

Subacute cardiotoxicity, consisting of acute myocyte damage and associated left ventricular dysfunction, occurs early during anthracycline therapy. We investigated the impact of myocardial dysfunction, defined herein by a shortening fraction (SF) < 29 % at any time during or after anthracycline therapy, on late onset cardiomyopathy and all-cause mortality, among childhood cancer survivors exposed to anthracyclines. In addition, we sought to identify subpopulations of subjects at highest risk for cardiomyopathy and death from all causes.

**Methods:**

Five hundred thirty-one childhood cancer survivors exposed to anthracyclines were enrolled and studied on average 10 (1.4–27.3) years following their initial exposure. The medical records were reviewed to identify known risk factors associated with cardiotoxicity, including cumulative anthracycline dose, length of post-therapy interval, administration of other cardiotoxic medications (vinca alkaloids), previous heart disease, radiation dose to the heart, history of bone marrow transplantation, age at treatment, gender, systolic dysfunction, and history of congestive heart failure during anthracycline therapy.

**Results:**

Ninety subjects (16.9 %) developed SF < 29 % and 71 patients (13.4 %) died on average 10 years after initial exposure (range 1.4–27.3 years). Total cumulative dose (OR 3.27, 95 % CI 1.94, 5.49, *p* < 0.001) and bone marrow transplantation (OR 2.57, 95 % CI 1.24, 5.30, *p* = 0.01) were found to be statistically significant risk factors for development of myocardial dysfunction. There was a 3-fold increase in the odds of having a SF < 29 % at any point during or following cancer therapy if a subject underwent bone marrow transplantation or had a total cumulative dose anthracycline therapy ≥ 240 mg/m^2^. The all-cause mortality ratio was almost seven-fold higher (95 % CI, 2.40-fold to 17.81-fold higher) if a subject developed systolic dysfunction, defined by a previous SF < 29 % anytime during or after anthracycline therapy. Nine deaths (12.7 %) were attributed to cardiovascular disease. The risk of dying as a result of cardiac disease also was significantly higher in individuals who had a SF < 29 % at any time during or after therapy.

**Conclusions:**

This study demonstrates an almost seven-fold increase in all cause mortality in pediatric cancer survivors with a history of anthracycline induced myocardial dysfunction defined as SF < 29 %.

## Background

More than half of the 380,000 long-term childhood cancer survivors in the United States face significantly increased risk for anthracycline induced cardiac morbidity and mortality. Despite the known cardiotoxic effects of these drugs [1], 50 % of the 12,400 children (<20 years) diagnosed with cancer each year receive anthracyclines as an essential component of their treatment protocols [2, 3]. The occurrence of cardiotoxicity is directly associated with the cumulative dose of anthracycline and increases over time [4–11], with morphological changes on myocardial biopsy noted in virtually all patients treated with doses of >240 mg/m^2^ [4]. Exposure to 250 mg/m^2^ or more of anthracyclines increases the relative hazard of congestive heart failure (CHF) 2–5 times, when compared to unexposed long-term survivors and has a five-fold increase in cardiovascular related mortality when compared to the general population [12].

Overall childhood cancer survivors experience 11 times the number of expected deaths than the general population [13]. Although the standardized mortality rate (SMR) declines with increase in follow-up and attained age, significant excess mortality persists in this patient population. The purpose of this study was to investigate the impact of anthracycline induced myocardial dysfunction, as defined by a SF < 29 %, on late onset cardiomyopathy and overall mortality in childhood cancer survivors. In addition, we sought to identify subpopulations of subjects at the highest risk for cardiomyopathy and death from all causes.

## Methods

This study was approved by the Connecticut Children’s Medical Center Institutional Review Board. All subjects were identified through a registry of pediatric cancer patients treated with anthracyclines between 1985 and 2013. The registry provides retrospective data regarding medical and cardiac history, chemotherapy, stem cell and radiation therapy, retrospective and prospective cardiac imaging and serologic biomarkers from pediatric cancer patients treated at CCMC. Subjects met inclusion criteria if they had a pediatric cancer diagnosis, received anthracyclines as part of their chemotherapy; and were ≤ 21 years at time of diagnosis. These subjects are followed at Connecticut Children’s Medical Center with yearly physical examinations and periodic echocardiograms based on age at treatment, radiation dose and cumulative anthracycline dose [14]. The medical records of all enrolled subjects were reviewed to identify known risk factors associated with cardiotoxicity, including cumulative anthracycline dose, length of post-therapy interval, administration of other potentially cardiotoxic medications (vinca alkaloids), pre-existing heart disease, radiation dose to the heart, bone marrow transplant, age at diagnosis, gender, systolic dysfunction, and history of congestive heart failure during anthracycline therapy. For analytic purposes, specific chemotherapeutic agents were grouped into anthracycline chemotherapeutic agents and vinca alkaloids [15]. We used the following formulas to convert to doxorubicin isotoxic equivalents prior to calculating total cumulative anthracycline dose: doxorubicin: total dose × 1; daunorubicin: total dose × 0.833; idarubicin: total dose × 5; mitoxantrone: total dose × 4 [14].

### Cause of death information

The vital status of each subject was ascertained as of December 31, 2013. Names of individuals who were reported to have died plus individuals who were lost to follow-up (no vital status available) were included in a National Death Index (NDI) search for deaths between 1985 to 2013. Information obtained from the NDI and medical records was used to categorize the cause of death as (1) a direct consequence of the original cancer diagnosis, (recurrent or progressive disease), (2) death due to cardio-toxicity and (3) non–treatment-related causes, such as deaths due to other medical conditions or external causes (e.g., suicide, car accident).

Lost to follow up was defined as not having seen a cardiologist, oncologist or CCMC provider in the prior two years.

### Echocardiography

Retrospective echocardiographic, M mode parameters were obtained including left ventricular end diastolic diameter (LVIDd), end systolic diameter (LVIDs), interventricular septal thickness (IVSd), posterior wall thickness (PWd), shortening fraction (SF), according to the American Society of Echocardiography recommendations for chamber quantification [16]. Shortening Fraction was obtained through retrospective review of echocardiograms that were performed as part of routine outpatient and inpatient clinic follow-up. For echocardiograms performed prior to April 1996, historic echo reports were abstracted with regards to reported M Mode parameters. We used fractional shortening as a measure of overall left ventricular systolic performance, as it has historically been the standard of care for assessment of myocardial performance in cancer treated patients exposed to anthracycline therapy and available on all patients starting in 1985. We defined systolic dysfunction as shortening fraction <29 % as this cut-off has been widely used as the threshold to withhold further Anthracycline chemotherapy in COG protocols. For analysis of global systolic dysfunction, SF < 29 % was included if obtained at any point during or after cancer therapy.

Persistent cardiomyopathy was defined as SF < 29 % in the most recent available echocardiogram in patients with a previous history of SF < 29 %, need for heart failure therapy (treatment with Carvedilol or ACE-inhibitors) being on the transplant waiting list or status post-heart transplant.

Cardiac outcomes studied include pericarditis, global systolic dysfunction defined as SF < 29 %, cardiomyopathy, CHF, and need for cardiac transplantation.

## Statistical analysis

Subjects’ values from medical record review and TTE were combined in a single study database. Group differences were compared using t-tests or their non parametric analogues for continuously distributed variables, chi-square and exact tests were utilized for categorical variables such as gender and previous SF < 29 %. Univariate logistic regression was used to select statistically significant risk factors for cardiotoxicity against the outcomes of SF < 29 % and all-cause mortality. These statistically significant risk factors became covariates in the multiple logistic regression model for each outcome. Kaplan-Meier survival analysis was used to demonstrate the effect of previous myocardial dysfunction on survival. All tests were two-tailed, and *p* < 0.05 was considered to indicate a statistically significant difference. SPSS software (IBM, Armonk, New York) was used for statistical analysis.

## Results

### Study population

**Fig. 1:**
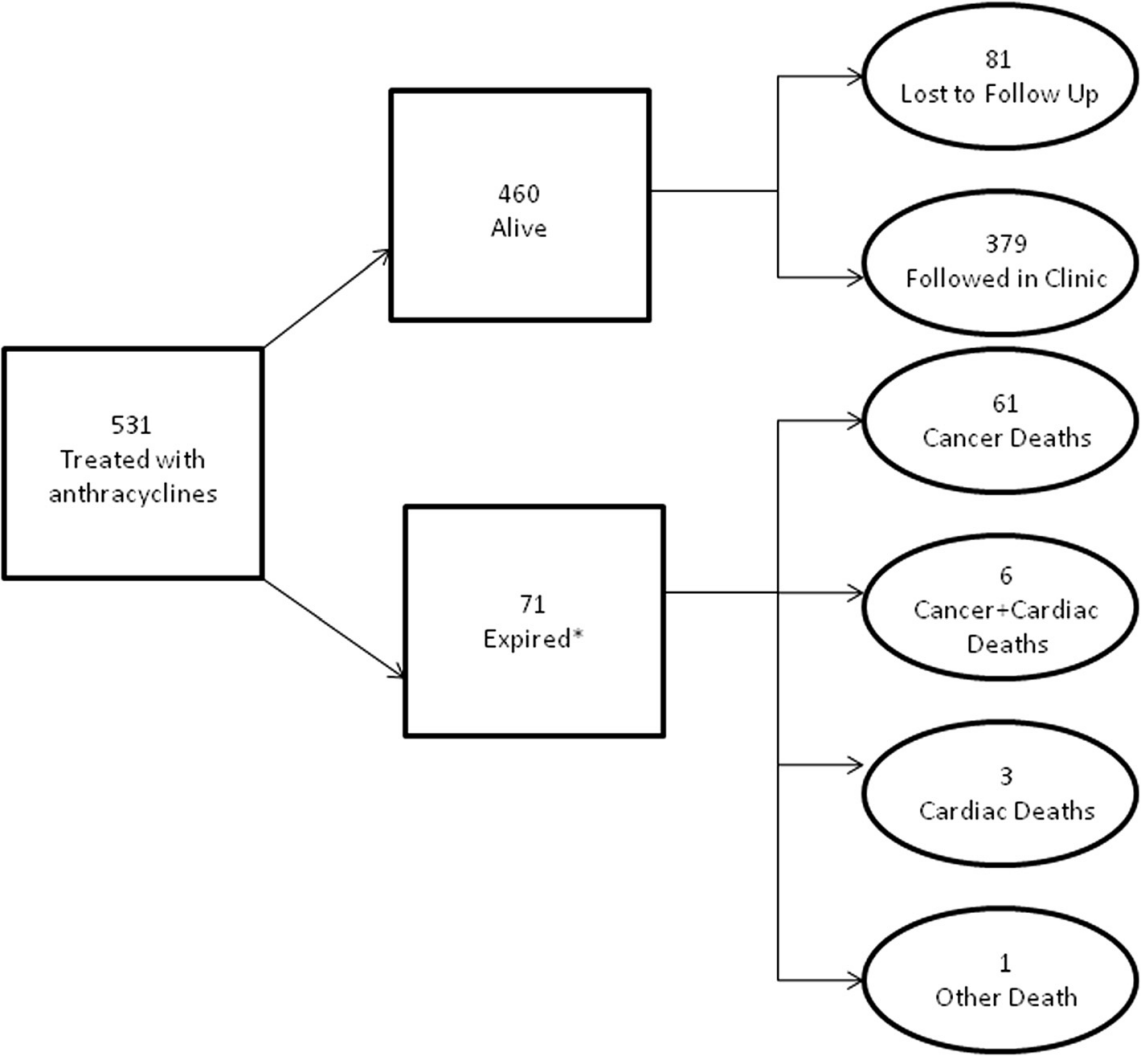
Cohort assignment. *Cause of death could be ascertained in 65 of 71 deaths from the National Death Index (NDI): 49 cancer deaths, 9 cardiac deaths, and 7 other deaths

A total of 531 subjects were identified through the cancer registry, of which 460 were alive on December 31, 2013 (Fig. 1). Eighty-one patients were lost to follow-up, and 379 were followed regularly at the late effects clinic. Demographic and Clinical Characteristics are presented in Table 1. Demographic and treatment characteristics were compared between the 379 patients currently followed in our registry and the 81 patients lost to follow-up. The 81 patients lost to follow up were significantly more likely to be female, (58 vs. 45%, *p* < 0.05) older at first diagnosis, (36 vs. 55 % less than 6 years, *p* < 0.01) and more likely to receive higher total cumulative doses of anthracyclines (31 % ≥ 240 mg/m^2^ vs. 18 %, *p* < 0.05) than the 379 patients actively followed in our registry. The most frequent type of cancer diagnosis in these subjects was acute lymphoblastic leukemia (ALL) in 175 (38 %), Hodgkin’s Lymphoma in 51 (11 %) and Non-Hodgkin’s Lymphoma in 37 (8 %). The mean cumulative anthracycline dose was 196 mg/m^2^, and 118 study subjects received a dose ≥ 240 mg/m^2^. The mean post-chemotherapy interval was 10 years (range 1.4 to 27.3 years). Sixty-seven subjects (12.6 %) received radiation to the chest and 424 (79.8 %) patients received vinca alkaloids as part of their chemotherapeutic regimen. Forty-nine (9.2 %) subjects had a history of previous bone marrow transplantation. Thirty (5.6 %) had heart disease diagnosed prior to the onset of chemotherapy. Classification of cause of death was possible in all 71 deaths when using information available through NDI and medical records.

**Table 1:** Demographic and clinical characteristics of cohort

	Alive	Expired
Characteristics	(*n* = 460)	(*n* = 71)
Sex		
Male	244	44
Female	216	27
Age at first cancer diagnosis,^a^ years		
0–2	111	11
3–6	126	13
7–10	65	8
11–15	86	27
> 15	64	8
Survival time after diagnosis,^a^ years		
0–5	72	52
6–10	142	10
11–15	102	3
> 15	136	2
Total cumulative dose anthracyclines,^b^ mg/m^2^		
5–158	238	20
159–240	104	7
241–390	74	14
≥ 400	18	14
Radiation Therapy to the Chest		
Yes	56	11
No	404	60
First cancer diagnosis		
Leukemia	230	27
Lymphoma	108	6
Solid Tumor	117	37
Unknown diagnosis	5	1

^a^Date of diagnosis only confirmed on 452/460 alive and 67/71 expired subjects

^b^Total cumulative dose information only confirmed on 434/460 alive and 55/71 expired subjects

### Cardiac related morbidity

**Fig. 2:**
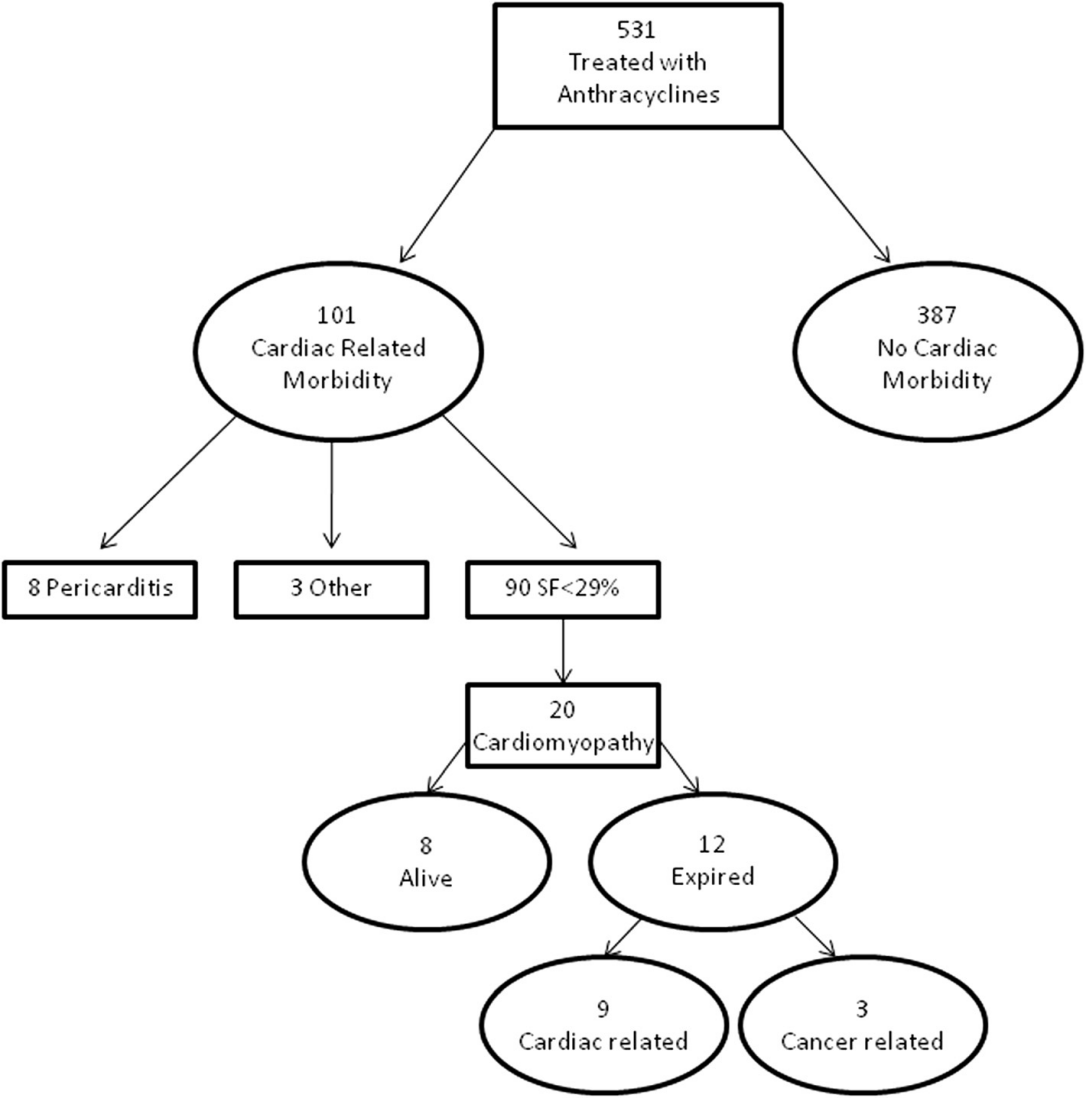
Cardiac morbidity and mortality among study population

**Fig. 3:**
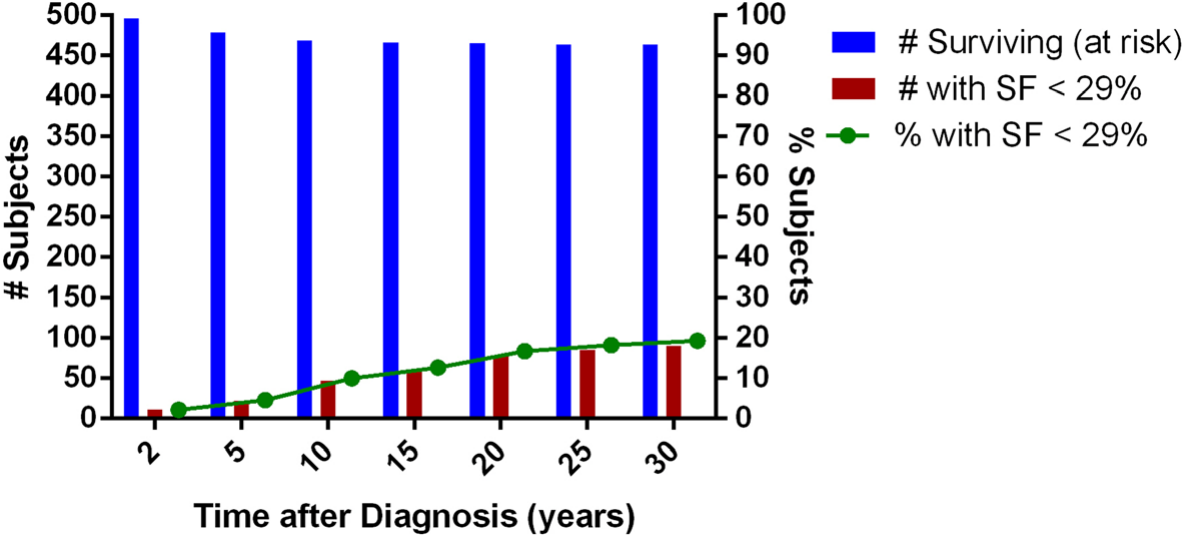
Development of SF<29 % over time. Time post Dx - yrs (2, 5, 10, 15, 20, 25, 30), Surviving at risk - n (496, 479, 469, 466, 465, 464, 464), SF < 29 % - n (11, 22, 47, 59, 78, 78, 90), % with SF < 29 % - 2.2, 4.6, 10, 12.7, 16.8, 18.3, 19.4

Cardiac related morbidity occurred in 101 of 531 subjects (19 %) (Fig. 2). Eight subjects (1.5 %) developed acute pericarditis during chemotherapy, 90 subjects (16.9 %) developed SF <29 % at any point during or after chemotherapy, and three additional subjects (0.6 %) presented with other cardiac findings. Eleven (2.2 %) of 496 survivors developed SF <29 % during chemotherapy, 22 (4.6 %) 5 years post diagnosis, 47 (10 %) 10 years, 59 (12.7 %) 15 years, 78 (16.8 %) 20 years and 85 (18.3 %) at 25 years (Fig. 3). Myocardial dysfunction was significantly associated with younger age at diagnosis, cumulative anthracycline dose greater than 240 mg/m^2^, bone marrow transplant and previous heart disease On multivariate analysis (Table 2), total anthracycline cumulative dose greater than 240 mg/m^2^ (OR 3.27, 95 % CI: 1.94, 5.49, *p* < 0.001) and bone marrow transplant (BMT), (OR 2.57, 95 % CI: 1.24, 5.30, *p* = 0.01) were found to be statistically significant risk factors for the development of myocardial dysfunction. There was a 3-fold increase in the odds of having a SF < 29 % at any point during therapy or at follow-up if a subject underwent BMT or had a total cumulative anthracycline dose greater than 240 mg/m^2^.

**Table 2:** The effect of risk factors on development of myocardial dysfunction (SF < 29 %) – Univariate and multiple logistic regression analysis

Risk Factors^a, b^	Unadjusted OR	95 % CI	*p*-value	Adjusted OR	95 % CI	*p*-value
Years post-chemo	0.99	0.96, 1.00	0.87	---	---	---
Age at Diagnosis	1.04	1.00, 1.08	0.04	1.00	0.96, 1.05	0.99
Gender	1.16	0.74, 1.83	0.51	---	---	---
Cumulative dose	3.36	2.06, 5.49	<0.001	3.27	1.94, 5.49	<0.001
Radiation to chest	1.82	0.99, 3.33	0.05	1.50	0.76, 2.96	0.24
Vinca Alkaloids	0.80	0.46, 1.37	0.41	---	---	---
BMT	2.98	1.57, 5.64	0.001	2.57	1.24, 5.30	0.01
Previous heart disease^c^	2.09	1.05, 4.15	0.04	1.87	0.85, 4.12	0.12
Cardio-protective drugs	1.86	0.80, 4.32	0.15	---	---	---

^a^Risk factors for cardiotoxicity include increased length of post-chemotherapy interval (years), younger age at diagnosis, female gender, total cumulative dose ≥240 mg/m^2^, radiation therapy to the chest, treatment with vinca alkaloids, bone marrow transplant, previous heart disease, non-use of cardio-protective drugs

^b^The 5 significant risk factors upon univariate analysis were selected as covariates for the multiple logistic regression model (younger age at diagnosis, total cumulative dose anthracyclines > 240 mg/m^2^, radiation to the chest, BMT and previous heart disease)

^c^Previous heart disease defined as: presence of congenital heart disease, pericardial effusion/tamponade, SVC syndrome, myocardial dysfunction prior to chemotherapy

A total of 20/90 subjects (22 %) with systolic dysfunction, defined as a shortening fraction < 29 %, had persistent cardiomyopathy. Of these twenty subjects, one subject underwent cardiac transplantation and one is awaiting transplant, three have died as a direct consequence of cardiac disease and in 6 additional subjects cardiomyopathy was as a contributing factor to their death. The remaining nine subjects continue to be followed at the heart failure clinic. Radiation to chest, BMT and previous heart disease, were significantly associated with persistent cardiomyopathy. On multivariate analysis BMT (OR 4.17, 95 % CI: 1.37, 12.69, *p* = 0.01) and previous heart disease (OR 6.04, 95 % CI: 2.10, 17.34, *p* < 0.001) were found to be statistically significant risk factors for the development of cardiomyopathy.

### Mortality data

Seventy-one of 531 registry subjects (13 %) have died (Fig. 2). Based on chart review and confirmation by the National Death Index, there were 59 deaths as a direct consequence of the original cancer diagnosis, including deaths due to recurrent or progressive disease, two deaths due to subsequent or secondary cancers, nine deaths due to cardiotoxicity and one death due to other medical conditions. On multivariate analysis, cumulative dose ≥240 mg/m^2^ (OR: 3.17, 95 % CI: 1.14, 8.85, *p* = 0.03), previous SF < 29 % (OR: 6.54, 95 % CI: 2.40–17.81, *p* < 0.001), BMT (OR: 5.22, 95 % CI: 1.57–17.37, *p* = 0.007), increased length of post-chemotherapy interval, (OR: 0.62, 95 % CI: 0.54–0.71, *p* < 0.001), and solid tumor diagnosis (OR: 4.13, 95 % CI: 1.72–9.87, *p* = 0.001), were found to be statistically significant risk factors for mortality (Table 3). There was over a five-fold increase in the odds of death if a subject had a BMT, and almost a seven-fold increase if a subject experienced systolic dysfunction as indicated by a previous SF < 29 %. Nine subjects died as a result of cardiovascular disease. The risk of dying as a result of cardiovascular disease was significantly higher in individuals who had previous shortening fraction < 29 % (*p* < 0.01) or previous heart disease (*p* < 0.05). Among the high dose group, excess risk of overall mortality was associated with a higher cumulative dose of anthracyclines, (434 ± 157 vs. 344 ± 81, *p* < 0.01) previous SF < 29 %, and history of bone marrow transplantation.

**Table 3:** The effect of risk factors on all-cause mortality – Univariate and multiple logistic regression analysis

Risk Factors^a, b^	Unadjusted OR	95 % CI	*p*-value	Adjusted OR	95 % CI	*p*-value
Years post-chemo	0.62	0.54, 0.70	<0.001	0.62	0.54, 0.71	<0.001
Age at Diagnosis (yrs)	1.05	1.01, 1.10	0.02	0.94	0.87, 1.02	0.12
Gender	0.69	0.42, 1.16	0.16	---	---	---
Cumulative dose	3.77	2.12, 6.71	<0.001	3.17	1.14, 8.85	0.03
Radiation to chest	1.32	0.66, 2.67	0.43	---	---	---
Vinca Alkaloids	0.32	0.19, 0.54	<0.001	0.37	0.13, 1.08	0.07
Previous SF < 29 %	4.52	2.62, 7.79	<0.001	6.54	2.40, 17.81	<0.001
BMT	4.21	2.19, 8.09	<0.001	5.22	1.57, 17.37	0.007
Previous heart disease	1.66	0.76, 3.61	0.20	---	---	---
Cardio-protective drugs	0.71	0.21, 2.39	0.58	---	---	---
Solid Tumor Diagnosis	3.2	1.94, 5.42	<0.001	4.13	1.72, 9.87	0.001

^a^Risk factors for cardiotoxicity include increased length of post-chemotherapy interval (years), younger age at diagnosis, female gender, total cumulative dose ≥240 mg/m^2^, radiation therapy to the chest, treatment with vinca alkaloids, previous shortening fraction < 29 %, bone marrow transplant, previous heart disease, non-use of cardio-protective drugs, solid tumor diagnosis

^b^The 7 significant risk factors upon univariate analysis were selected as covariates for the multiple logistic regression model (increased length post-chemotherapy interval, younger age at diagnosis, total cumulative dose anthracyclines > 240 mg/m^2^, use of vinca alkaloids, previous SF < 29 %, BMT, and solid tumor diagnosis)

**Fig. 4:**
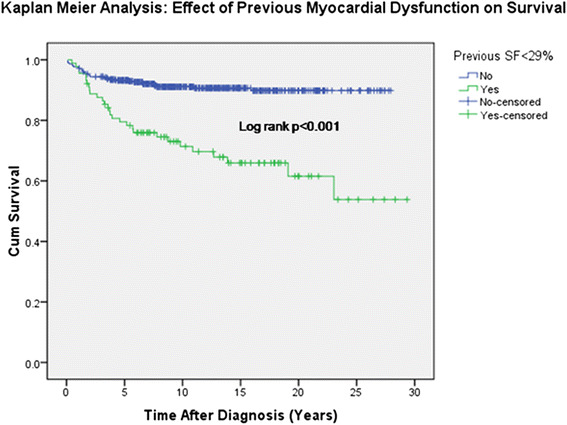
Kaplan-Meier analysis: Effect of previous myocardial dysfunction on survival

Cumulative survival was 88 % at 10 years from diagnosis, 85 % at 15 years, 84 % at 20 years, and 82 % at 25 years. Individuals with an original diagnosis of leukemias and lymphoma had the best overall survival, with 87 % of leukemia subjects and 94 % of lymphoma subjects surviving at 20 years. Individuals with an original diagnosis of solid tumors had the poorest overall survival, with 70 % survival at 20 years. Cumulative survival was significantly lower (log rank *p* < 0.001) in the subjects with SF < 29 % with cumulative survival of 71 % at 10 years, 66 % at 15 years, 62 % at 20 years and 54 % at 25 years (Fig. 4).

Of the 531 subjects in the registry, 30 were treated with cardio-protective medication (dexrazoxane). Subjects in the dexrazoxane group received a higher total cumulative anthracycline dose (314.2 ± 163.2 vs.189.2 ± 112.7 mg/m^2^, *p* < 0.001). Three subjects died of non-cardiac related deaths. One subject treated with dexrazoxane had pericarditis, 1 had a heart transplant due to primary cardiomyopathy (not AIC related), and 8 developed SF <29 %. Two of these subjects went on to develop persistent cardiomyopathy, and are still alive at last date of follow-up.

## Discussion

Our findings indicate that long term pediatric cancer survivors with evidence of anthracycline induced myocardial dysfunction, defined as a history of SF <29 %, experienced a seven fold increase in risk of death. In addition, subjects with a history of SF <29 % had a cumulative survival of 54 % at 25 years, compared to 85 %, in the group with no previous myocardial involvement. Other factors, including increased length of post chemotherapy interval, younger age of diagnosis, total cumulative anthracycline dose, history of BMT and a solid tumor diagnosis, were also associated with increased risk for all-cause mortality. The association between myocardial dysfunction and all cause mortality including cancer related deaths is a novel finding in this patient population and brings attention to the adverse cumulative effect of comorbidities among patients living with chronic diseases. The effect of comorbidity on adverse outcomes has been previously described in patients with cancer and heart failure. The risk of cancer and non cardiovascular related mortality was found to be higher in heart failure (HF) compared with HF-free subjects by Hasin et al. [17]. Furthermore, the presence of overt cardiomyopathy may limit the options for additional chemotherapy with anthracyclines or with protocols containing cardiotoxic agents such as the use of biologic targeted therapies (e.g., sorafenib), a common exclusion criteria on clinical trials. Further studies will be needed to examine the mechanisms of this association.

The increased risk of cardiomyopathy among survivors of childhood cancer, who already experience significant excess mortality, underscores the importance of surveillance for subclinical myocardial dysfunction in this patient population. Recent consensus guidelines by the American Society of Echocardiography (ASE) and the European Association of Cardiovascular Imaging (EACVI) advocate the use of standard transthoracic echocardiographic techniques in addition to 2D strain and 3D imaging acquisition for the diagnosis of Cancer Therapeutics–Related Cardiac Dysfunction (CTRCD) in adult patients during and after cancer therapy. In addition, cardiac magnetic resonance imaging is emerging as an important technique for detection of subclinical and overt cardiotoxicity given its excellent reproducibility for assessment of left ventricular volume, mass and function and unique capability for tissue characterization of the left and right ventricular myocardium [18–21]. Several serum biologic soluble markers including N-terminal pro-BNP (NT-pro-BNP), cardiac troponin T (cTnT) and cardiac troponin I (cTnI) have been found to be useful markers in the early detection of AIC [22, 23]. Implementation of a multimodality imaging approach including the use of serologic biomarkers to best identify subclinical myocardial dysfunction, may allow clinicians to detect structural myocardial abnormalities prior to the development of heart failure. Furthermore, given the inherent differences between pediatric and adult patients exposed to anthracycline therapy, guidelines for multimodality imaging in children for routine surveillance of patients should be established.

The period of subclinical cardiotoxicity is characterized by structural and functional myocardial abnormalities and is highly prevalent amongst asymptomatic pediatric cancer survivors [21, 24–28]. Treatment of subclinical dysfunction with Enalapril and possibly Carvedilol may help prevent or decrease the incidence of cardiac events if started earlier after chemotherapy [29, 30] and in addition may decrease cancer progression and cancer specific mortality in some patients [31]. Furthermore, the use of dexrazoxane is able to reduce cardiac injury associated with the use of doxorubicin [22, 32]. Future studies designed to assess the effect of early implementation of these therapeutic strategies on all cause mortality may help clarify this important question.

Consistent with previous studies, preexisting heart disease, age at diagnosis, cumulative anthracycline dose, and bone marrow transplant were significant risk factors associated with myocardial dysfunction [33, 34]. Persistent cardiomyopathy occurred in 22 % of subjects with previous myocardial dysfunction, and in agreement to previous reports, was associated with a survival of < 50 % at 5 years [35]. Previous heart disease, radiation to the chest and BMT were significant risk factors associated with persistent cardiomyopathy [12].

Our specific cause of mortality is similar to that observed in the Childhood Cancer Survivor Study (CCSS) [36], and the British Childhood Cancer Survivor Study [13]. Recurrence of cancer was the most common cause of death, followed by treatment-related consequences and secondary or subsequent cancers. In a large cohort of childhood cancer survivors, the risk of dying as a result of cardiac disease was significantly higher in individuals who received a cumulative anthracycline dose of greater than 360 mg/m^2^ and who received radiation dose that exceeded 5Gy [12]. In addition, our findings indicate that anthracycline induced myocardial dysfunction increases the risk of death from all causes as well as the risk of dying as a result of cardiac disease.

We have assembled an interdisciplinary team of cardiologists, and oncologists to implement primary secondary and tertiary heart failure prevention strategies in childhood cancer survivors exposed to cardiotoxic chemotherapy [37]. With the goal of reducing the variability in clinical practice and improving outcomes, our team has developed a management tool in which the different interventions by each sub-specialist involved in the patient’s care is defined, optimized, and mapped out over time. The creation of this interdisciplinary team has facilitated the implementation of prevention strategies and development of clinical pathways of therapy designed to standardize clinical care among multiple subspecialists caring for these patients.

### Study limitations

Shortening fraction was considered until recently as the standard of care for estimation of global systolic function in the longitudinal evaluation of patients exposed to anthracycline therapy and included widely in COG protocols. It is now considered that shortening fraction should not be used, since it only takes into account the anterior septum and inferolateral wall for the calculation of EF [18]. We chose SF given its availability in all patients since 1985. Since starting our cardio-oncology program five years ago we have incorporated a comprehensive echocardiographic evaluation based on ASE guidelines [18].

## Conclusion

Our study demonstrates that patients with myocardial dysfunction, defined by a SF < 29 % at any time during or after anthracycline therapy had a lower cumulative survival. Through the interdisciplinary collaborations between cardiology and oncology, and development of evidence-based guidelines, geared towards early detection and treatment of subclinical cardiotoxicity clinicians may improve overall survival in this vulnerable population.

## Abbreviations

3DE: 3-dimensional echocardiography
AIC: anthracycline induced cardiotoxicity
ALL: acute lymphoblastic leukemia
BMT: bone marrow transplant
CHF: congestive heart failure
DTI: doppler tissue imaging
IVSd: interventricular septal thickness
LVIDd: left ventricular end diastolic diameter
LVIDs: left ventricular end systolic diameter
NDI: National death index
PWd: posterior wall thickness
SF: shortening fraction
SMR: standardized mortality ratio
STE: speckle-tracking echocardiography
